# Engaging in a dialogue with the Luton Roma community on access to healthcare services and co-producing solutions

**DOI:** 10.3389/fpubh.2026.1716479

**Published:** 2026-03-30

**Authors:** Nasreen Ali, Britzer Paul Vincent, Osasenaga Akenbor, Fiona Mackay, Erica Cook, Crina Morteanu, Gurch Randhawa

**Affiliations:** 1 Institute for Health Research, University of Bedfordshire, Luton, United Kingdom; 2 School of Psychology, University of Bedfordshire, Luton, United Kingdom; 3 Luton Roma Trust, Luton, United Kingdom

**Keywords:** access to health, barriers, community engagement, co-production, ethnicity, Luton Roma community, qualitative research, United Kingdom

## Abstract

**Background:**

Luton, a super-diverse town in the East of England, is home to one of the largest Roma populations in the UK. Roma communities experience significant health inequalities, particularly in accessing healthcare services, contributing to poorer health outcomes. However, there is limited research on effective ways to engage Roma communities and address these disparities. This study aimed to explore the views of the Luton Roma community on accessing healthcare services and to co-develop solutions.

**Methods:**

A qualitative study was conducted using eleven focus groups with 64 participants. Discussions were transcribed verbatim and analysed using Thematic Framework Analysis.

**Findings:**

Three main themes emerged. First, key health concerns included maternity and child healthcare, dissatisfaction with emergency care, long waiting times, and perceived quality of care. Second, barriers to access comprised limited knowledge of services, language difficulties, the need to travel to Romania for care, and perceived discrimination from providers. Third, suggested improvements focused on raising awareness of services, providing language support, and ensuring culturally competent, compassionate care. Gender and age shaped healthcare experiences. Women emphasised caregiving responsibilities and communication challenges, while men highlighted work-related pressures and system-level issues. Younger participants faced digital literacy challenges, middle-aged adults navigated caregiving demands, and older participants reported cumulative discrimination and distrust.

**Conclusion:**

Improving healthcare access for Roma communities requires systemic, rights-based interventions addressing language, discrimination, and structural obstacles, rather than expecting individuals to adapt. Policy actions should include professional interpretation, culturally competent care, anti-discrimination mechanisms, and active Roma involvement in service design. Improving health-seeking behaviours among Roma communities requires attention to age and gender dynamics, as socially constructed gender roles and life-course experiences shape health perceptions, caregiving responsibilities, and engagement with healthcare services. Community-led models, such as Roma health mediators, are essential for building trust, bridging communication gaps, and promoting equitable health outcomes.

## 1 Background

One of the largest concentrations of the Roma community in the UK is in Luton (1), a super-diverse town in the East of England. The Roma community come from all over Eastern Europe, from countries such as Romania, Bulgaria, the Czech Republic, and Slovakia (2), and comprises Europe’s largest ethnic minority, widely recognised as a socially vulnerable population (3). However, research has highlighted challenges in identifying the population size of the Roma community and the limited availability of data on their health profiles, partly because some individuals are reluctant to disclose their identities due to stigma associated with the term *‘*Roma’ and the lack of citizenship and/or official documentation (4). While the term Roma refers to the range of groups including Gypsies, Travellers, Sinti, Bargees/Boat dwellers and New Age Travellers with diverse histories, cultural, and linguistic backgrounds, there are also debates about its meaning across different contexts. For example, while a significant proportion of Roma communities across Europe regard the term ‘gypsy’ as offensive, the term is still used quite often in the UK (4).

In the UK, the Roma community experience poorer health outcomes compared to other ethnic minority groups, with reports of lower life expectancy, increased suicide rates, poor infant and child health, a higher burden of both communicable and non-communicable diseases and lower self-reported health alongside higher mortality risk (4). These health inequalities are shaped not only by individual behaviours or barriers to healthcare but also by broader social determinants, including poverty, limited education, inadequate housing, unemployment, and social exclusion. Operating cumulatively across the life course, these structural factors compound disadvantage and contribute to persistently poorer health outcomes among the Roma population (5, 6). Evidence from European Roma populations shows that these socio-economic determinants are strongly associated with worse self-reported health, greater chronic disease burden, and increased vulnerability to illness compared with neighbouring non-Roma populations, even when demographic differences are taken into account, highlighting the need for multi-sectoral policy responses that address structural disadvantage rather than healthcare alone (6). Research further indicates that intersecting inequalities such as discrimination, insecure employment, and inadequate living conditions restrict access to essential services and contribute to persistent disparities in health and well-being among Roma communities in the UK and beyond (4). Recognising these determinants reinforces the need for healthcare access research to extend beyond service provision and consider wider social, economic, and political contexts that shape health and engagement with care.

In addition, gender inequalities in Roma health arise from the interaction between their social roles and cultural norms and wider structural factors such as ethnic discrimination, poverty, and social exclusion (4–6). Together, these forces expose Roma women and men to different risks across the life course, leading to distinct pathways of disadvantage and ill health (7).

Roma women consistently report poorer self-rated health, higher rates of depression, obesity, and reproductive health burdens linked to early marriage, repeated caregiving, low education, and restricted labour-market access (8). They also experience institutional discrimination and stigma, which can delay care-seeking and increase psychosocial stress (9). In contrast, Roma men face elevated risks from hazardous informal work, substance use, and premature mortality, but masculine norms discourage help-seeking and the use of health services (10). Together, these differences in life-course exposures create gendered patterns of health risk within Roma communities.

The poor health outcomes of the Roma community are linked to challenges in accessing and engaging with healthcare services, including barriers such as language, communication, mistrust of health services, difficulties in registering with GPs, and lack of access to timely, quality and affordable healthcare (4, 11). This evidence aligns with findings from previous studies highlighting issues such as lower uptake of immunisation among the Roma community (11). Collectively, this highlights the need for research focused on reducing health inequalities among Roma communities and for targeted interventions to improve access and engagement to healthcare services (4, 12).

The Luton Roma Trust was established in 2015 and provides a wide range of free services to the Luton Roma community, facilitating integration into wider society whilst respecting and maintaining their cultural values (1, 13). Responding to the Roma community’s needs, the Luton Roma Trust has identified that access to healthcare remains one of the most significant challenges faced by the Roma community in Luton (14). A recent evaluation carried out by the Luton Roma Trust shows that the Roma community experiences high levels of mistrust towards statutory authorities and faces multiple barriers to accessing healthcare services. This is evidenced by extremely low vaccination uptake, with over 96% of the Roma living in Luton reported as unvaccinated (14).

The existing evidence base highlights persistent gaps in cultural competence and understanding of Roma communities within the UK health services, which can result in shortcomings in service provision and negative health outcomes (15). Although studies in the UK have explored barriers to accessing healthcare services, there remains a dearth of evidence on the nuanced social dynamics and underlying structural influences shaping interactions between members of the Roma community and healthcare service providers (16, 17). These interactions are further shaped by the interplay of migration histories, discrimination, and environmental contexts that influence the Roma community’s experiences of accessing healthcare services (18).

Given the vulnerabilities of this community, this study adopts a participatory approach to engage directly with members of the Luton Roma community to explore experiences of accessing healthcare services. It addresses a clear gap in the UK evidence base by foregrounding community perspectives and examining social and structural factors that shape engagement with healthcare. Therefore, we engaged with the Roma community to better understand their views on healthcare access and co-develop solutions that can inform more equitable and culturally responsive health service provision.

## 2 Materials and methods

### 2.1 Study design and approach

This study used a qualitative research methodology to enable direct engagement with the Luton Roma community and to understand how healthcare access is experienced in practice. This approach allowed participants to describe their experiences, concerns and priorities in their own terms, which was central to understanding barriers to access and opportunities for improvement. The study sought to engage in a dialogue with the Luton Roma community to better understand their views on healthcare access and co-develop solutions that can inform more equitable and culturally responsive health service provision (19, 20).

Focus group discussions were used to create space for shared reflection on everyday interactions with health services (20), including issues of trust, communication, discrimination and cultural expectations that shape decisions about care-seeking. By focusing on participants’ accounts of real-world experience, the study identified how barriers to healthcare access arise and are navigated in daily life, supporting the co-development of relevant and contextually grounded solutions.

The Talk, Listen, Change (TLC) approach is an ethnographic community engagement model designed to recruit and work with participants who are often overlooked in research and service design (21). This approach is grounded in the recognition that conventional recruitment methods tend to privilege already-visible voices, thereby reproducing existing inequalities in whose experience informs research and policy. Rather than relying on the most visible or frequently consulted individuals, the approach prioritises inclusion across ethnicity, age, sex, linguistic, national and social class differences, recognising the heterogeneity of lived experience and avoiding homogenisation of Roma identities (21). In doing so, the TLC approach positions recruitment not as a procedural step, but as a critical methodological intervention that shapes whose knowledge is produced and valued.

Community Researchers who were bilingual, both male and female, and embedded within local communities facilitated recruitment and data collection. Their positionality enabled access to social networks and trust that are not readily available to external researchers, allowing engagement with individuals who may not participate in conventional research due to language barriers, mistrust of services, or cultural and religious considerations and who are more accurately described as ‘lesser heard’ rather than ‘hard to reach’ (21–23). This deliberate engagement strategy strengthens the validity of the findings by ensuring that accounts reflect lived experiences that are typically absent from research, service evaluations and needs assessments.

The effectiveness of participatory approaches rests on the extent to which they move beyond tokenistic inclusion to engage lesser-heard voices, highlighting recruitment practices as a critical methodological concern (21). Within this research, the TLC approach operationalised participation by embedding inclusion, trust, and accessibility at the point of recruitment and facilitation, rather than treating participation as an outcome alone. Being an insider can enhance contextual insight but also risks introducing assumptions into interpretation (24, 25). To address this and achieve necessary analytical estrangement, we employed reflective journaling (26, 27), peer debriefing (28), and systematic coding procedures (24, 25) to ensure that interpretations were grounded in participants’ accounts rather than researcher expectations. Acknowledging our positionality through reflexive practices strengthened the credibility and confirmability of our qualitative findings.

Focus groups were conducted in familiar and accessible settings and facilitated in participants’ preferred language, allowing individuals to share experiences and perspectives in ways that felt safe and meaningful. This approach prioritised voice and lived experience, generating rich qualitative data on barriers to healthcare access and potential solutions within the local context (21).

The term ‘Roma community’ is used in this paper to reflect a diverse range of ethno-national, linguistic and religious groups originating from Central and Eastern Europe (29, 30). To avoid generalisation, recruitment and engagement were conducted with sensitivity to self-defined identities, including individuals who identify as Roma but may not align with Office for National Statistics ethnic categories (31–33). This approach enabled participation by individuals with limited English proficiency and those less visible in official data, ensuring that the study reflected the diversity of Roma communities.

### 2.2 Study setting

The study took place in Luton, a super-diverse local authority and one of the most ethnically diverse areas in the UK, reflecting a vibrant mix of cultures, and home to one of the largest Roma populations in the UK (1). Focus group discussions with the Roma community took place at the premises of the Luton Roma Trust, and the data collection was completed between June and September 2023.

### 2.3 Study participants

The study included Roma men and women living in Luton, with participants aged between 16 and 60 years. Men made up a slightly larger proportion of the sample (56%), while women accounted for 44%. Most participants reported having no formal educational qualifications, including 83.3% of men and 92.9% of women. In terms of employment status, nearly half of the participants (47%) were unemployed. A further 38% were in either part-time or full-time employment, while 9.4% described themselves as self-employed. Most respondents (87.5%) lived with members of their immediate family. Smaller numbers reported living with extended family (6.3%), living alone (4.7%), or other household arrangements (1.6%). Housing conditions varied, with just over half of participants (52%) residing in two-bedroom properties. Nearly a quarter (23%) lived in homes with three bedrooms, one-fifth (20%) occupied one-bedroom accommodation, and a small proportion (5%) lived in properties with four or more bedrooms. This breadth of demographic and socio-economic characteristics was facilitated by the TLC approach, which enabled engagement with a diverse range of Roma community members.

### 2.4 Data collection

A purposive sampling (20) technique was used to invite individuals from the Roma community living in Luton to participate in the focus group discussions. The Luton Roma Trust provided essential support to identify a suitable bilingual Roma community researcher to recruit and facilitate the focus group discussions, enabling the removal of cultural, linguistic, and religious barriers and supporting the inclusion of ‘lesser heard voices’. The Roma Trust facilitated access to participants through their established community networks. We tailored our TLC branding (21) to develop publicity materials designed to support recruitment, strengthen engagement, and build trust within the community. Participants were asked to complete a socio-demographic biographic sheet to help contextualise the findings (as presented in section 2.3). Focus group discussions were conducted using a topic guide developed specifically for members of the Roma community. The topic guide was designed to facilitate open discussion while ensuring consistency across groups and covered three core thematic areas. These included participants’ knowledge and understanding of healthcare services available; their experiences of accessing and using these services, including factors that supported access, positive encounters, challenges, and negative experiences; and participants’ views on how access to healthcare and overall experiences could be improved for Roma people, with particular emphasis on reducing barriers and adverse experiences. The flexible structure of the topic guide allowed participants to elaborate on issues of importance to them while enabling the research team to explore shared and divergent perspectives systematically.

With consent, the focus group discussions were conducted in Romanian, audio-recorded, translated and transcribed by a bilingual researcher. In total, eleven focus group discussions were conducted, comprising 64 participants (Table 1), which is a substantial sample for a qualitative study using focus group discussions, and data saturation was achieved at the level of codes and themes (34). The focus group discussions were segregated by age and sex to promote open discussion and freedom of expression (35) (Table 1). The duration of the focus group discussion ranged between 45 min and 70 min.

**Table 1:** Characteristics of the focus group participants (*n* = 64).

S.no	FG code	Sex	Religion	Age group	Language	No. of participants
1	FG1	Male	Christian	26–35 years	Romanian	6
2	FG2	Female	Christian	26–35 years	Romanian	5
3	FG3	Female	Christian	46–55 years	Romanian	5
4	FG4	Female	Christian	16–21 years	Romanian	6
5	FG5	Male	Christian	16–21 years	Romanian	6
6	FG6	Female	Christian	36–45 years	Romanian	6
7	FG7	Female	Christian	56+ years	Romanian	5
8	FG8	Female	Christian	22–25 years	Romanian	7
9	FG9	Male	Christian	46–55 years	Romanian	6
10	FG10	Male	Christian	22–25 years	Romanian	6
11	FG11	Male	Christian	56+ years	Romanian	6

FG, focus group.

### 2.5 Ethics approval

This study was approved by the Institute for Health Research Ethics Committee at the University of Bedfordshire (IHREC1003). Participation in the study was entirely voluntary. Members of the TLC community research team provided prospective participants with an information sheet and obtained verbal or written consent (via email) for contact regarding participation in a focus group. Written consent was obtained before the commencement of any focus group discussion. Participants were allowed a minimum of 1 week to review the information. Following their involvement, all participants received a debrief sheet containing details of relevant support services and helplines.

All data were handled confidentially and stored securely at the University of Bedfordshire. Audio recordings and transcripts from interviews and focus groups were anonymised, and care was taken to ensure that no participant could be identified through personal details such as names or identifiable location data (including IP addresses or postcodes).

### 2.6 Data analysis

A Thematic Framework Analysis approach was used to analyse the data (36). This involved a systematic familiarisation with the data, identification of the key themes to form a coding frame, indexing the material according to the coding framework, and interpreting the findings in relation to research, policy and practice considerations. Data saturation was achieved at the level of codes and themes (37, 38).

### 2.7 Trustworthiness of the data

This study upheld several key principles of qualitative research trustworthiness, as articulated in established methodological literature on rigour and quality in qualitative inquiry. Credibility was enhanced through prolonged engagement with the Roma community, the use of bilingual, culturally embedded community researchers, and the careful facilitation of focus groups in participants’ preferred language, ensuring that the findings reflected participants’ lived experiences and perspectives rather than researcher assumptions (39). Dependability and confirmability were supported by systematic documentation of the research process, transparent description of methods, and structured thematic analysis, allowing the study’s procedures and analytic decisions to be clearly traced and audited as grounded in participant data rather than researcher bias (40). Transferability was facilitated by rich contextual description of the setting, recruitment, and participant characteristics, enabling readers to assess the wider applicability of findings to similar contexts (41). Reflexivity, recognised as integral to transparency, was embedded in the study through culturally sensitive design, recruitment, and interpretation, with explicit acknowledgement of researcher positionality and its influence on data generation and analysis (42). Together, these practices align with well-recognised criteria for establishing trustworthiness in qualitative research and strengthen the rigour, transparency and credibility of the study’s insights.

## 3 Findings

Three main themes emerged regarding healthcare experiences among the Roma community. Firstly, the main health conditions causing concern for the Roma community included maternity and child healthcare, dissatisfaction with emergency care, long waiting times, and the perceived quality of care. Secondly, barriers that prevent Roma people from accessing healthcare include limited knowledge of services, language difficulties, the need to travel to Romania for care, and perceived discrimination from providers. Finally, suggestions for improving access to healthcare focused on increasing awareness of available services, providing language support, and ensuring culturally competent and compassionate care.

### 3.1 The main health conditions causing concern for the Roma community

#### 3.1.1 Types of health conditions causing concern to Roma people

Participants from four of the focus group discussions discussed how maternity and child healthcare, along with adult healthcare, were inadequate and how Roma people were treated less favourably compared to other ethnic groups. This was a cause of concern. One male participant from a focus group discussion described:

They [i.e., Doctors] do not look after our children here the way doctors do in Romania. I had a case with one of my children, and she was not looked after. All the doctors here did was look at the child for a bit, and that was it; he [Doctor] sent us home saying there was nothing wrong (FG9, Male, aged 46–55).

#### 3.1.2 Emergency healthcare was much more frequently mentioned, and participants were generally dissatisfied with the quality of this care

Participants from ten of the focus group discussions identified maternal, infant, and child health, as well as adult health, as their primary healthcare priorities. When seeking care for these concerns at hospital emergency departments, they reported experiencing long waiting times and expressed overall dissatisfaction with the quality of care received. One of the female participants from the focus group discussion mentioned:

I’ve been to the hospital many times with my child, in the emergency room, and the people from the reception made me wait for hours… [You] are made to wait for hours, even if the children are vomiting, feverish, screaming, and screaming in pain. The even bigger problem is when the receptionists give the excuse that there are not enough people to take care of the emergencies at that moment or that there are no doctors available. It does not seem normal to me (FG6, Female, aged 36–45).

### 3.2 Barriers that prevent Roma people from accessing healthcare

#### 3.2.1 Knowledge of healthcare services

Participants from eight of the focus group discussions did not know about the range of available healthcare services. A male participant mentioned:

I personally have not heard about these services. I have not had anyone to teach me or give me information about them (FG10, Male, aged 22–25).

While participants from three focus group discussions knew about the services, they were not sure how to seek help for preventative services. One of the male participants reported:

We heard about these services, but we don’t know how to access them (FG9, Males, aged 46–55).

Participants often did not know how to refer to preventative healthcare, and they reported struggling to secure GP appointments. One of the female participants said:

I call the emergency room and try to make an appointment [for gynaecology] … I don’t know where else to call (FG4, Female, aged 16–21).

#### 3.2.2 Reasons for going ‘back home’ for healthcare

Participants from nine of the focus group discussions consistently reported a strong preference for travelling to Romania for healthcare. They explained this was because of long waiting times and the quality of care they received. One of the male participants mentioned:

I am not satisfied with the health services… because most of the time these services are unprofessional. The quality of services is poor, and from what I have observed, treating patients with superficiality and indifference is another big problem (FG5, Male, aged 16–21).

#### 3.2.3 Language and communication barriers

Language and communication barriers were reported by participants across all eleven focus group discussions. The primary obstacles to accessing healthcare for Roma participants arose from limited English proficiency and the lack of access to healthcare professionals or interpreters who could speak Romanian. These barriers led to difficulties and delays in arranging appointments, extended waiting times before being seen, and challenges in communicating effectively during consultations. Language and communication difficulties were particularly highlighted when interactions took place via telephone, as mentioned by one female participant as follows:

When I want to make a GP appointment, I have to wait a very long time, I am handed from operator to operator [over the phone], I am asked a lot of meaningless questions, and it doesn’t help me at all (FG2, Female, aged 26–35).

In four of the focus group discussions, participants told us that problems in accessing healthcare were exacerbated when staff gave them electronic devices to check into an appointment, make an appointment, or book a translator, because they did not include a Romanian language option and therefore many reported not being able to use the devices offered to accomplish these intended tasks. One of the female participants described this experience as follows:

I have a problem with getting GP appointments. We Roma people don’t speak or read English. We are not given a translator to be able to communicate better with people in the health services in Luton, and when I try to call to get an appointment at the GP, they redirect me to their webpage to make the appointment online. I don’t know how to navigate the internet, I can’t manage on my own to get an appointment at the GP (FG6, Female, aged 36–45).

Furthermore, among the five focus group discussions, language and communication barriers led participants to feel fear, confusion, and helplessness during critical medical emergencies, hindering their ability to understand the situation. For example, one female participant described her experience during an emergency involving her newborn as follows:

I did not have the benefit of an interpreter; my newborn baby was blue, vomiting and seemed limp, weak. I got very scared and started screaming for help. No one was touching my baby, no one explained to me what was going on, everyone just told me to stay calm, that there was no problem with the baby. I asked for a translator, I asked to allow my husband to enter the room, because he speaks and understands English, but they refused, they just told me to wait for a translator to come and explain everything. I waited for hours, and finally the baby was taken and put in an incubator, but the translator was still not given to me (FG6, Female, aged 36–45).

#### 3.2.4 Perceived discrimination from service providers

Almost all participants from all the focus group discussions reported feeling they were treated differently and less favourably, on the grounds of being Roma and how they were referred to. One of the male participants said:

The problem is that we Roma are called Romanian Gipsy (FG11, Male, aged 56+).

Participants from four of the focus group discussions said they preferred to be called Roma, and not ‘gypsy’ or ‘gipsy’, which they consider insulting and also disliked the term ‘traveller’. Two participants commented that being Roma was equated to being called a ‘gipsy’ or ‘gypsy’ by people, when providing a detailed account about their perceptions of being treated differently and less favourably, due to being Roma. One of the male participants said:

There are differences between our ethnicity and other types of ethnicities. Always when we go to the GP, we wait for hours to be picked up, the same when we try to make GP appointments (FG10, Male, aged 22–25).

Participants from all the focus group discussions linked discrimination to not being able to speak English. In addition to this, participants also discussed their experience of discrimination in relation to other ethno-national groups. One of the male participants said:

I have noticed that they prefer to treat those who have the same ethnicity as theirs first and then treat the rest of us Roma (FG11, Male, aged 56+).

A male participant in another focus group continued to say the following:

I have definitely been discriminated against because I don’t know how to explain those [my] symptoms in English, and I can’t find a person there who can understand me or help me in that case (FG1, Male, aged 26–35).

Participants perceived discrimination when they asked for a translator at the hospital for better communication. A male participant reflected his feeling of being discriminated against because language barriers were not accommodated, staff ignored his questions, and he perceived exclusion and negative attention while they spoke in their own language, leading him to feel unfairly treated. He said:

I asked for information about the child’s health, and she kept telling me in English to wait. Every time I asked her a question, she always replied that I had to wait. They would just tell me to wait and speak amongst themselves in their native language, not English. It didn’t seem right; I had the impression that they were talking about me because their eyes were always directed towards me when they were talking to each other… I expected them to help me with a translator, not to have to wait an hour and a half for nothing. Why didn’t they take the necessary attitude to do their best to understand what I wanted to say and what my problems were? It didn’t seem fair at all (FG10, Male, aged 22–25).

Another participant from a younger age group focus group mentioned how a lack of access to online platforms has been a challenge for her to access the service. She said:

Not being of age means I cannot access the online platform (FG4, Female, aged 16–21).

However, throughout the focus group, participants did not express concern in relation to religious stigma or discrimination.

### 3.3 Suggestions for improving access to healthcare in Luton

#### 3.3.1 The need for more information on the range, referral routes to and location of healthcare services

Participants from all eleven focus group discussions mentioned the need for information on the range, referral routes to and location of healthcare services. They suggested that information should be provided in Romanian. One of the male participants suggested:

There are no people to help inform people about health services in a way that we understand. I come here to a foreign country, I don’t know the language, and I don’t know where to go or who to go to for more information about what I need (FG10, Male, aged 22–25).

Suggestions for the best ways to provide this information included leaflets and flyers, videos, the internet, posters, GPs, schools, discussion groups, information by telephone and post. Participants said all information should be provided in Romanian or other languages they understand. One of the male participants said:

I think that information about health services… should be provided through videos and leaflets in Romanian (FG10, Male, aged 22–25).

#### 3.3.2 Tackling issues of language and communication, including the lack of availability of translators

Among the participants from six focus group discussions, there was a consensus about the need for support for tackling language and communication issues by providing translated information. Participants also emphasised the importance of having translators available to address the Romanian English language barrier. A male participant said:

To provide a translator, or access to a telephone translator, or to provide information through the Roma communication centres or videos in Romanian, would be very helpful to us (FG10, Male, aged 22–25).

#### 3.3.3 Culturally competent and compassionate care from healthcare professionals

Participants from three focus group discussions suggested that the term ‘gipsy’ should not be used on forms, and that healthcare professionals needed training to be more culturally competent and compassionate towards the Roma community. A male participant said:

The problem is that we Roma are called Romanian Gipsy… and we do not want to be called so, which leads to discrimination (FG11, Male, aged 56+).

## 4 Discussion

This study presents the views of the Luton Roma community on accessing healthcare and co-producing solutions. The main healthcare conditions causing concern for the Roma community were maternity and child healthcare, and adult healthcare. Participants did not discuss chronic health conditions, such as diabetes and obesity, which we specifically asked about. This absence may indicate that immediate and acute healthcare needs take precedence over longer-term condition management in the context of structural barriers to access. Our participants were most likely to access GP surgeries and emergency services. Overall, participants were dissatisfied with their experiences of using healthcare services, particularly in relation to emergency care, waiting times, and communication.

Differences were identified in the information obtained from focus group participants according to gender and age. Women more frequently discussed issues related to maternity, childbirth, and child health, and their accounts often included more detailed and emotionally charged descriptions of negative experiences, particularly in emergency and neonatal care. They also placed greater emphasis on communication difficulties, the lack of interpreters, and the distress associated with not understanding clinical decisions affecting their children. In contrast, men tended to focus more on system-level concerns, such as long waiting times, perceived unprofessionalism, and dissatisfaction with the overall quality of healthcare services, often drawing comparisons with healthcare provision in Romania. These differences between men and women illustrate how their social roles, caregiving responsibilities, and differences in life-course experiences (i.e., access to education, work, exposure to the world, emotional make-up) could shape their health perceptions, barriers, and access, with women emphasising challenges related to caregiving, emotional burden, and communication while men focus more on work-related and system-level issues (7–9).

Age-related differences were also apparent: younger participants often described barriers related to digital literacy and navigating online appointment systems and difficulties understanding healthcare administrative systems, compounded by lower educational attainment and social exclusion, which limit access to guidance and support (43). Middle-aged adults frequently prioritise caregiving responsibilities in their interactions with healthcare services because they are often the primary carers for children, older adult relatives, or extended family. This role shapes their healthcare needs and perspectives, as time constraints, financial pressure, and family obligations determine when and how they access services (10). Older Roma emphasised experiences of long-term discrimination and social marginalisation, where cumulative exposure to ethnic stigma, poverty, and exclusion across the life course shaped their perceptions of healthcare institutions. Their repeated negative experiences reinforced distrust and heightened their sensitivity to inequities in treatment (4). These findings should be interpreted with caution, as the study was not designed to conduct a comparative analysis by sex, gender, or age, and the number of participants in each subgroup was limited. Nonetheless, these patterns indicate that healthcare barriers are not experienced uniformly and suggest the need for age- and gender-responsive approaches to service design and delivery.

We identified multiple barriers, which included limited knowledge about the range and referral routes to healthcare services in Luton, long waiting times and quality of care, which resulted in decisions to return to Romania for healthcare. Language and communication problems were a significant barrier and resulted in difficulties and delays in setting up health appointments, delays in being seen, and difficulties in communication during appointments. Language barriers, therefore, emerged as a foundational mechanism through which exclusion from healthcare services was reproduced across multiple points of contact with health systems.

Our findings are similar to another study among the Roma community in Leeds, UK, which concluded that the poor health outcomes of Roma people were closely linked to language barriers, which prevented accessing healthcare and healthcare messages, as well as the wider social determinants, including housing, employment opportunities and money (17). Research with the Roma people in Europe also highlights the poor health outcomes experienced by Roma people compared to the majority populations (3). Poor health outcomes have been associated with the marginalisation and exclusion of the Roma people, and the problems that occur when accessing healthcare (44, 45). A systematic review of ‘Gypsy’, Roma and ‘traveller’ access and engagement with health services, reviewed ninety-nine studies from 32 countries, reported barriers to health service usage were related to the organisation of health systems, discrimination, culture, language, health literacy, service-user attributes and economic barriers (10, 46). A study with the Roma people in Spain argued that barriers could be summarised under two categories, which were existing institutional arrangements and interaction with health professionals (47). What this study adds is detailed, place-based insight into how these barriers are experienced in everyday encounters with UK health services and how they inform decisions to disengage or seek care elsewhere.

Discussion with the Roma community highlighted several actionable strategies to improve healthcare access. This included the importance of increasing information on the available healthcare services and referral routes, improving health literacy on preventative healthcare services, health and wellbeing, and chronic conditions, recruiting and training community members, as well as health and wellbeing champions to transfer knowledge and information, and increasing language and communication support for the Roma community. These recommendations point to the need for interventions that operate at both service and community levels, rather than placing responsibility on individuals to navigate inaccessible systems. Studies have also shown that the role of intermediaries, such as Roma health mediators, plays a key role in bridging communication gaps, providing health education, facilitating access, and building trust between healthcare systems and the Roma population (48–50).

The Roma community participants emphasised the importance of raising awareness that the preferred identifier is Roma, rather than “gipsies,” “gypsies,” or “travellers,” alongside the need for cultural competency training for professionals. Research suggests that those labels represent one of the last socially tolerated forms of prejudice in Europe, and that anti-Gypsyism remains a significant barrier to the inclusion of Roma communities by reinforcing harmful stereotypes about Roma culture (4). Such discriminatory practices, including the use of stigmatising language and unequal treatment in healthcare settings, can undermine trust in services, contribute to disengagement and delays in seeking care, and ultimately exacerbate health inequalities (18, 51–53).

These findings can be best understood through the lens of the social determinants of health, which emphasises that health inequalities arise from the social conditions in which people are born, grow, live, work, and age, rather than from individual behaviour alone (54). Language barriers, in particular, intersect with other structural determinants, limiting individuals’ ability to navigate healthcare systems, understand referral pathways and communicate effectively with healthcare professionals. Experiences of discrimination and stigma further compound these barriers, reinforcing social marginalisation and mistrust in health services. From a human rights perspective, these experiences raise concerns regarding the right to the highest attainable standard of health, non-discrimination, and equitable access to healthcare, as articulated in international frameworks including the International Covenant on Economic, Social and Cultural Rights (55) and reinforced by the World Health Organisation (56). Together, these findings indicate that improving healthcare access requires structural changes that address language access, discrimination, and system design, rather than expecting Roma communities to adapt to inadequately inclusive healthcare systems (54, 57).

The language and communication challenges align with wider research demonstrating that limited proficiency in the dominant language of the country where an individual lives is often the first and most significant barrier to accessing healthcare across Europe (58) and is closely associated with poorer access, reduced service use, and worse health outcomes among migrant populations (59). Participants’ reliance on informal strategies, such as family members or digital tools to mediate communication, reflects broader trends in the growing use of translation technologies in healthcare contexts. While tools such as machine translation can offer immediate support, recent research cautions that they may also introduce risks related to accuracy, confidentiality, and power imbalances, particularly when used as substitutes rather than supplements for professional interpretation (60, 61).

Improving healthcare access for Roma communities, therefore, requires rights-based, structural interventions that go beyond cultural awareness alone, including guaranteed access to professional interpretation, anti-discrimination accountability mechanisms within health services, and the meaningful involvement of Roma communities in the co-design of services. Evidence suggests that community-led approaches, such as Roma health mediators and participatory service design, can reduce institutional barriers, improve trust, and promote more equitable healthcare experiences (6, 62). Addressing these intersecting determinants is essential to shifting responsibility away from individuals towards health systems that actively uphold equity, dignity, and rights.

This study provides a broad perspective on access to healthcare for the Luton Roma community and their lived experiences.

To the best of our knowledge, this is the first study exploring access to healthcare services with the Luton Roma community, where the researcher was also from the same community. This study provides in-depth contextualised findings and co-produced solutions to ensure services meet the needs of the Luton Roma community. Furthermore, this study used a qualitative approach, allowing us to build trust with members of the Roma community in Luton, gain an in-depth understanding of their lived experiences in accessing healthcare services and co-design solutions. However, while our findings may be comparable to those from other multi-ethnic settings and healthcare systems, the qualitative research approach prioritises depth and richness of data rather than generalisability, which is an inherent characteristic of this type of research (63). Importantly, the TLC approach and purposive recruitment strategy ensured inclusion of participants who are typically excluded from research due to cultural, linguistic or religious barriers, strengthening the relevance and credibility of the findings for service improvement (21, 64).

## 5 Conclusion

The findings from our research provide a clear evidence base for policymakers and decision-makers to prioritise structural interventions aimed at improving healthcare access for Roma communities. Addressing language barriers, discrimination, and system-level obstacles requires a shift away from approaches that place responsibility on individuals to navigate complex healthcare systems. The study also highlights that community-led initiatives, such as Roma health mediators or community connectors, which bridge communication gaps, strengthen trust between communities and services, and support more effective engagement with healthcare systems. Improving health-seeking behaviours among Roma communities also requires attention to age and gender dynamics, as socially constructed gender roles and life-course experiences shape their health perceptions, caregiving responsibilities, and engagement with healthcare services. Ultimately, reducing inequalities in healthcare access from Roma communities will require coordinated action across health systems, social policy, and community engagement, ensuring that services address structural determinants of health, promote inclusion, and uphold the right to equitable healthcare.

## Data Availability

The original contributions presented in the study are included in the article/supplementary material, further inquiries can be directed to the corresponding author.
